# WNT7A Promotes EGF-Induced Migration of Oral Squamous Cell Carcinoma Cells by Activating β-Catenin/MMP9-Mediated Signaling

**DOI:** 10.3389/fphar.2020.00098

**Published:** 2020-02-26

**Authors:** Hui Xie, Yadong Ma, Jun Li, Huixia Chen, Yongfu Xie, Minzhen Chen, Xuyang Zhao, Sijie Tang, Shuo Zhao, Yujie Zhang, Jun Du, Feimin Zhang, Luo Gu

**Affiliations:** ^1^ Jiangsu Key Lab of Oral Diseases, Afﬁliated Hospital of Stomatology, Nanjing Medical University, Nanjing, China; ^2^ Department of Implantology, Changzhou Stomatological Hospital, Changzhou, China; ^3^ Department of Physiology, Nanjing Medical University, Nanjing, China; ^4^ Department of Stomatology, Affiliated Hospital of Jiangsu University, Zhenjiang, China; ^5^ Department of Biochemistry and Molecular Biology, Nanjing Medical University, Nanjing, China; ^6^ Department of Pathology, The People’s Hospital of Bozhou, Bozhou, China

**Keywords:** EGF, WNT7A, AKT, migration, OSCC

## Abstract

**Aims and hypothesis:**

Epidermal growth factor (EGF) has been shown to induce the migration of various cancer cells. However, the underlying signaling mechanisms for EGF-induced migration of oral squamous cell carcinoma (OSCC) remain to be elucidated. WNT7A, a member of the family of 19 Wnt secreted glycoproteins, is commonly associated with tumor development. It is mostly unknown whether and, if so, how EGF modulates WNT7A in OSCC cells. The role of WNT7A in OSCC was thus investigated to explore the underlying signaling mechanisms for EGF-induced migration of OSCC.

**Methods:**

Cell migration was measured by Wound healing assay and Transwell assay. Western blotting was carried out to detect the expression of WNT7A, MMP9, β-catenin, p-AKT, and p-ERK. The cells were transfected with plasmids or siRNA to upregulate or downregulate the expression of WNT7A. The location of β-catenin was displayed by immunofluorescence microscopy. Immunohistochemistry was carried out to confirm the relation between WNT7A expression and OSCC progression.

**Results:**

The present study showed that the levels of WNT7A mRNA and protein were increased by EGF stimulation in OSCC cells. Besides, it was proved that p-AKT, but not p-ERK, mediated the expression of WNT7A protein induced by EGF. Furthermore, the inhibition of AKT activation prevented the EGF-induced increase of WNT7A and matrix metallopeptidase 9 (MMP9) expression and translocation of β-catenin from the cytoplasm to the nucleus. Moreover, histological analysis of OSCC specimens revealed an association between WNT7A expression and poor clinical prognosis of the disease.

**Conclusions:**

The data in this paper indicated that WNT7A could be a potential oncogene in OSCC and identified a novel PI3K/AKT/WNT7A/β-catenin/MMP9 signaling for EGF-induced migration of OSCC cells.

## Introduction

It is well known that Wnt signaling is one of the most important pathways, playing an essential role in a variety of cellular processes (Ghosh et al., 2019; Goldsberry et al., 2019; Sharon et al., 2019). Historically, it includes two categories: the canonical and the non-canonical Wnt signalings. The Wnt/β-catenin pathway, known as the canonical Wnt signaling, controls cell growth, differentiation, apoptosis, and self-renewal (Rosenbluh et al., 2012; Gonzalez-Moles et al., 2014; Nusse and Clevers, 2017; Alamoud and Kukuruzinska, 2018). This pathway is aberrantly activated during the development of multiple cancers and can coordinate with other pathways to regulate cancer cell proliferation, migration, and invasion (Yu et al., 2012; Myant et al., 2013; Novellasdemunt et al., 2015; Rudy et al., 2016; Pohl et al., 2017; Kartha et al., 2018; Lee et al., 2019; Reyes et al., 2019).

WNT3A and WNT7B are known to activate the WNT/β-catenin pathway in colon cancer and pancreatic adenocarcinoma, respectively (Arensman et al., 2014; Qi et al., 2015). *WNT7A*, a member of the *Wnt* gene family, has been identified as an oncogene in pancreatic ductal adenocarcinoma and colon cancer (Thomas et al., 2003; Becer et al., 2019). The effect of WNT7A on cancer development is type-dependent. It can accelerate cancer cell proliferation and induce cancer progression through the canonical Wnt/β-catenin pathway in ovarian and endometrial cancers (Liu et al., 2013; MacLean et al., 2016). On the other hand, in non-small cell lung carcinoma (NSCLC) and gastric cancer (GC), WNT7A has been found to act as a tumor suppressor *via* non-canonical Wnt signaling (Avasarala et al., 2013a; Avasarala et al., 2013b; Liu et al., 2019). The role of WNT7A in oral squamous cell carcinoma (OSCC) is unclear, and this is the focus of our research.

The tumor microenvironment (TME) provides a distinct advantage in tumor-aggressive capability (Liubomirski et al., 2019). It has been documented that cancer cells may gain invasive and migratory properties when they receive TME signals such as EGF, VEGF, TNF-α, and TNF-β, which could promote tumorigenesis and metastasis (Dewangan et al., 2019; Lee, 2019; Lin et al., 2019). EGF is mainly synthesized by the salivary glands, making saliva a potential source of EGF in the oral environment (Bernardes et al., 2011). EGF has been shown to induce the migration of various cancer cells (Thomas et al., 2003; Tumur et al., 2015). Furthermore, EGF receptor (EGFR) is overexpressed in oral cancer tissues and is closely associated with the degree of malignancy of tongue cancer (Ansell et al., 2016; Sun et al., 2018). Previous studies have shown that there is an association between EGF/EGFR and the Wnt family. For example, it is reported that there is a crosstalk between Wnt and EGF signalings (Zhang et al., 2015; Liu et al., 2017) and that the over-expression of WNT10B can induce epidermal-keratinocyte transformation through activating the EGF pathway (Lei et al., 2015). However, despite these recent studies, it is still mostly unknown whether and, if so, how EGF modulates WNT7A-expression in OSCC cells.

It is generally accepted that tumor cell migration plays a vital role in tumor progression (Yamashita et al., 2017; Qin et al., 2018; Koedoot et al., 2019). In the present study, we identified WNT7A as a potential oncogene mediating EGF signaling and confirmed the role of AKT as a critical molecular connection between EGF stimulation and WNT7A expression in OSCC cells. Furthermore, we showed that WNT7A could activate Wnt/β-catenin signaling, which then increased MMP9 expression and led to cell migration. The results of this study clearly demonstrate a unique relationship between EGF signaling and WNT7A expression in regulating cancer cell migration, which could be essential in the identification of therapeutic targets for the treatment of OSCC.

## Materials and Methods

### Ethics Statement

All immunohistochemistry assays with human tumor specimens were conducted under the institutional guidelines of Jiangsu Province, China.

### Cell Culture

Human OSCC cell line HSC3 was purchased from the Cell Resource Center for Biomedical Research, Tohoku University (TKG0484) and CAL27 (CRL-2095) was purchased from the American Type Culture Collection (ATCC, Manassas, VA, USA). The cells were cultured in DMEM medium (Biological Industries, Bet-Haemek, Israel) supplemented with 10% fetal bovine serum (Gibco, Thermo Scientific, Grand Island, NY, USA) at 37°C with 5% CO_2_. The cells were serum-starved overnight, followed by EGF (R&D Systems, Minneapolis, MN, USA) treatment.

### Cell Transfection

Three WNT7A siRNAs were obtained from GenePharma (Shanghai, China); their sequences were 5’-GCGCAAGCAUCAUCUGUAATT-3’ (siRNA #1), 5’-CCGG-GAGAUCAAGCAGAAUTT-3’ (siRNA#2), and 5’ –CCACCUUCCUGAAGAUCA-ATT-3’ (siRNA #3), respectively. The full-length cDNA of Human WNT7A was cloned into a pcDNA3.1-HA-C vector (Youbio, Hunan, China). The cells were transfected with 25 nM siRNA using Lipofectamine 2000 (Invitrogen, Carlsbad, CA, USA). For plasmid transfection, FuGENE HD Transfection Reagent (Promega Corporation, Madison, USA) was used.

### Cell Viability Assay

The cells were seeded in 96-well plates at a density of 5×10^3^ cells per well. After 24 h, cells were serum-starved overnight and treated with EGF for 0, 12, 24, and 48 h. A commercial CCK8 assay kit (Dojindo Laboratories, Kumamoto, Japan) was used to detect the viability of the cells. The absorbance value was measured at a wavelength of 450 nm using a microplate reader (Bio-Tek, Vermont, American). The assay was performed in more than three independent experiments.

### Cell Proliferation Assay

Cell proliferation was analyzed with a 5‐ethynyl‐2′‐deoxyuridine (EDU) Kit (RiboBio, Guangzhou, China). The cells cultured in a 24-well plate were treated with or without EGF (20 ng/mL) for 24 h. After the addition of EDU, the cells were cultured for an extra 2 h. After the incubation, the cells were stained according to the manufacturer’s instructions. Briefly, the culture medium was discarded, and 4% paraformaldehyde was added to fix the cells at room temperature for 30 min. After the fixation, the cells were washed with a glycine solution (2 mg/mL) for 5 min in a shaker. After 0.5% Triton X-100 was added, the washing was continued for an extra 10 min. Then, the cells were washed two times with PBS. After the washing, 100 μL of 1 × Apollo^®^ reaction cocktail was added to each well, and the plate was incubated for 30 min. After the incubation, the cells were washed three times with 0.5% Triton X-100. Finally, the cells were stained with 100 μL of 1 × Hoechst 33342 for 30 min and washed three times with PBS. Photographs were collected using an inverted microscope (Carl Zeiss Meditec, Jena, Germany).

### Wound Healing Assay

The cells were seeded in a 6-well plate. When the cells reached confluence, the wound-healing assay was performed by scraping through the cell monolayer with a sterile P200 pipette tip. The plate was washed twice with PBS to remove non-adherent cells. Then, new medium was added, and the cells were cultured for 24 h. Photographs were collected by using an inverted microscope (Carl Zeiss Meditec, Jena, Germany) at 0 and 24 h time points.

### Transwell Migration Assay

Transwell assay was performed using a transwell plate (Millipore, Billerica, MA, United States). HSC3 cells were isolated, washed, and suspended in DMEM without FBS. The cells (2×10^5^) were seeded in the upper chamber, and the lower chamber was filled with 600 μL DMEM with 10% FBS. The cells were treated with U0126 or LY294002 in the absence or presence of EGF (20 ng/mL) for 24 h. Then, the cells remaining on the upper side of the membrane were wiped off with cotton swabs. The cells that had migrated onto the lower side of the membrane were fixed with 4% formaldehyde and stained with 0.1% crystal violet. Images of the migrated cells were taken with an inverted microscope (Carl Zeiss Meditec, Jena, Germany). The cells in three randomly selected regions of the field were counted using Image J software. The experiments were repeated at least three times.

### Total RNA Isolation and Quantitative Real-Time PCR

Total RNA was isolated using TRIzol reagent (Invitrogen, Carlsbad, CA, USA). The reverse transcription was carried out by HiScript II Q RT SuperMix (Vazyme, Nanjing, China). The quantity of target cDNAs was measured using the ABI StepOne™ Plus Real-Time PCR System (Applied Biosystems, Foster City, CA, USA) and analyzed with StepOne software v2.1 (Applied Biosystems, Foster City, CA, USA). The 2^−ΔΔCT^ method was used to calculate gene expression levels. Each sample was measured in triplicate relative to GAPDH values.

### Western Blotting

The protein sample was collected, and its concentration was measured by the BCA protein assay and normalized to equal amounts. The proteins were separated by 10% SDS-PAGE gels and transferred onto nitrocellulose membranes. The membranes were blocked with 5% skim milk in TBST for 1 h at room temperature and incubated with primary antibody overnight at 4°C. After incubation with a secondary antibody for 1 h at room temperature, the bands were visualized with ECL reagent (Millipore, Billerica, MA, USA) and analyzed using the Quantity One image analysis program (Bio-Rad).

### Immunofluorescence Microscopy and Immunohistochemistry

The cells grown on a glass coverslip were washed with PBS and fixed with paraformaldehyde. Permeabilization was done using 0.1% TritonX-100 before blocking in 1% BSA for 1 h at room temperature. The cells were incubated with primary antibody overnight, followed by incubation with an Alexa-coupled secondary antibody (Thermo Fisher Scientific) for 1 h at room temperature. DAPI (Southern Biotech, Birmingham, AL, USA) was used for staining cell nuclei. Pictures were acquired using an Olympus BX43 microscope (Olympus, Tokyo, Japan).

Oral cancer tissue microarrays were purchased from Outdo Biotech (Shanghai, China). OSCC and corresponding tissue samples were used for immunohistological staining in our study. The paraffin sections were de-paraffinized and hydrated. Later, peroxidase blocking was carried out with 3% H_2_O_2_ in methanol for 15 min at 37°C. The sections were incubated with primary antibody overnight and then with secondary antibody for 1 h. Afterward, these sections were incubated with DAB and counterstained with hematoxylin. Finally, the staining was analyzed by evaluating the percentage of the WNT7A positive cells and the staining intensity, allowing the assessment of an immunoreactivity score (IRS).

### Reagents and Antibodies

EGF was purchased from R&D Systems (Minneapolis, MN, USA). Phospho-ERK1/2, phospho-AKT (Thr308), phospho-AKT (Ser473), AKT, β-catenin, ERK1/2, and Histone3 antibodies were purchased from Cell Signaling Technology (Boston, MA, USA). MMP9 antibody was purchased from Bimake (Shanghai, China). GAPDH antibody and HRP-linked anti-rabbit secondary antibodies were purchased from Bioworld Technology (Louis Park, MN, USA). WNT7A antibody was purchased from Abcam (Cambridge, MA, USA). MEK inhibitor U0126 was purchased from Promega (Madison, WI, USA), and PI3K/AKT inhibitor LY294402 was purchased from Sigma (St. Louis, MO, USA).

### Statistical Analysis

Statistical analyses were performed using Prism 7.0 software (GraphPad Software, Inc., La Jolla, CA, USA). All experiments were repeated at least three times. Differences between two groups were analyzed by Student’s *t*-test. Repeated measures analysis of variance was used to compare the differences among multiple groups. *P* < 0.05 represents statistical significance, and *P* < 0.01 represents sufficiently statistical significance (two-tailed).

## Results

### EGF Induces the Migration of OSCC Cells

**Figure 1:**
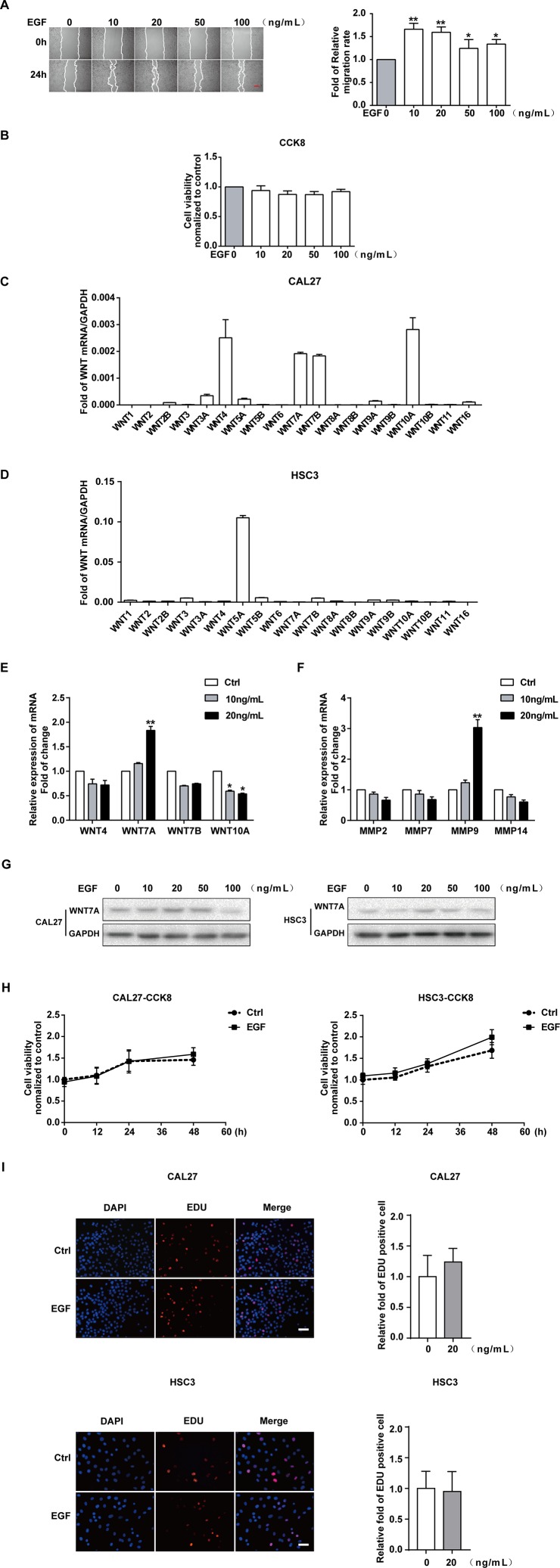
EGF induces the migration of OSCC cells. The results of the wound healing assay and quantification of migration rate **(A)**, showing that the cell migration was increased by EGF stimulation for 24 h. *P < 0.05, **P < 0.01. Scale bar, 100 μm. **(B)** The results of the CCK8 assay showed that treatment with the indicated concentration of EGF for 24 h did not noticeably affect the viability of CAL27 cells. **(C)** qPCR detection of WNT mRNA expression in CAL27 cells showed that WNT4, WNT7A, WNT7B, and WNT10A were abundantly expressed. **(D)** qPCR detection of WNT mRNA expression in HSC3 cells. **(E)** Among the highly expressed WNT mRNAs, the WNT7A mRNA level was doubled with EGF (20 ng/mL) treatment for 24 h. *P < 0.05, **P < 0.01. **(F)** The qPCR result, which showed the maximum change in MMP mRNA expression with EGF (20 ng/mL) stimulation for 24 h. **P < 0.01. **(G)** The results of Western blotting showed that WNT7A was remarkably increased after EGF (20 ng/mL) stimulation in both HSC3 and CAL27 cells. **(H)** The results of CCK8 assay in CAL27 and HSC3 cells showed that there was no significant difference between the two indicated groups at 0, 12, 24, and 48 h. **(I)** EDU assay showed that EGF (20 ng/mL) treatment for 24 h did not affect cell proliferation in CAL27 and HSC3 cells.

CAL27 cells were treated with different concentrations of EGF and underwent a wound-healing assay to examine the effect of EGF on the migration of the cells. The results showed that the migration of CAL27 cells was increased by EGF stimulation, especially with stimulation by 10 ng/mL and 20 ng/mL EGF (**Figure 1A**). Cell Counting Kit-8 (CCK8) assay was used to measure the viability of CAL27 cells incubated with EGF. The results showed that treatment with EGF did not noticeably affect the viability of CAL27 cells (**Figure 1B**). qPCR was applied to detect the mRNA expression of all members of the *Wnt* gene family in CAL27 and HSC3 cells (**Figures 1C, D**). The results showed that the mRNAs of WNT4, WNT7A, WNT7B, and WNT10A were abundantly expressed in the CAL27 cells and the mRNA of WNT5A was abundantly expressed in the HSC3 cells (**Figure 1C**). This difference might relate to the characteristics of different cell lines. Besides, the qPCR result showed that the mRNA level of WNT7A was remarkably increased after EGF stimulation (20 ng/mL, 24 h) (**Figure 1E**). Furthermore, the qPCR result showed that the expression of MMP9 mRNA was obviously increased under the stimulation with 20 ng/mL EGF (**Figure 1F**).

Taken together, we hypothesized that WNT7A might play a vital role in the EGF-induced migration of OSCC cells. Thus, we used HSC3 cells to verify our hypothesis. The results of Western blotting showed that WNT7A was remarkably increased after EGF stimulation (20 ng/mL, 24 h) in both HSC3 and CAL27 cells (**Figure 1G**).

Since FZDs, LRP, and EGFR play an essential role in the regulation of WNT signaling (Ma et al., 2017; Tripurani et al., 2018; Yin et al., 2018; Hsu et al., 2019), we checked the expression of their mRNAs in CAL27 and HSC3 cells (**Figures S1A–C**). The expression of EGFR, MMP9, and WNT7A was also detected with Western blotting in both CAL27 and HSC3 cells (**Figure S1D**). We found that FZD3, FZD6, LRP5, LRP6, and EGFR were highly expressed in the OSCC cells. To exclude the effect of cell viability on the migration, CCK8 was carried out to detect the viability of the cells with EGF (20 ng/mL) treatment at 0, 12, 24, and 48 h in CAL27 and HSC3 cells (**Figure 1H**). In addition, the EDU experiment was also used to exclude the effect of EGF (20 ng/mL) treatment on cell proliferation (**Figure 1I**). No significant differences in cell viability and proliferation were found. Therefore, 20 ng/mL of EGF was chosen as the optimal concentration in this research.

### WNT7A is Associated With the EGF-Induced Migration of OSCC Cells

**Figure 2:**
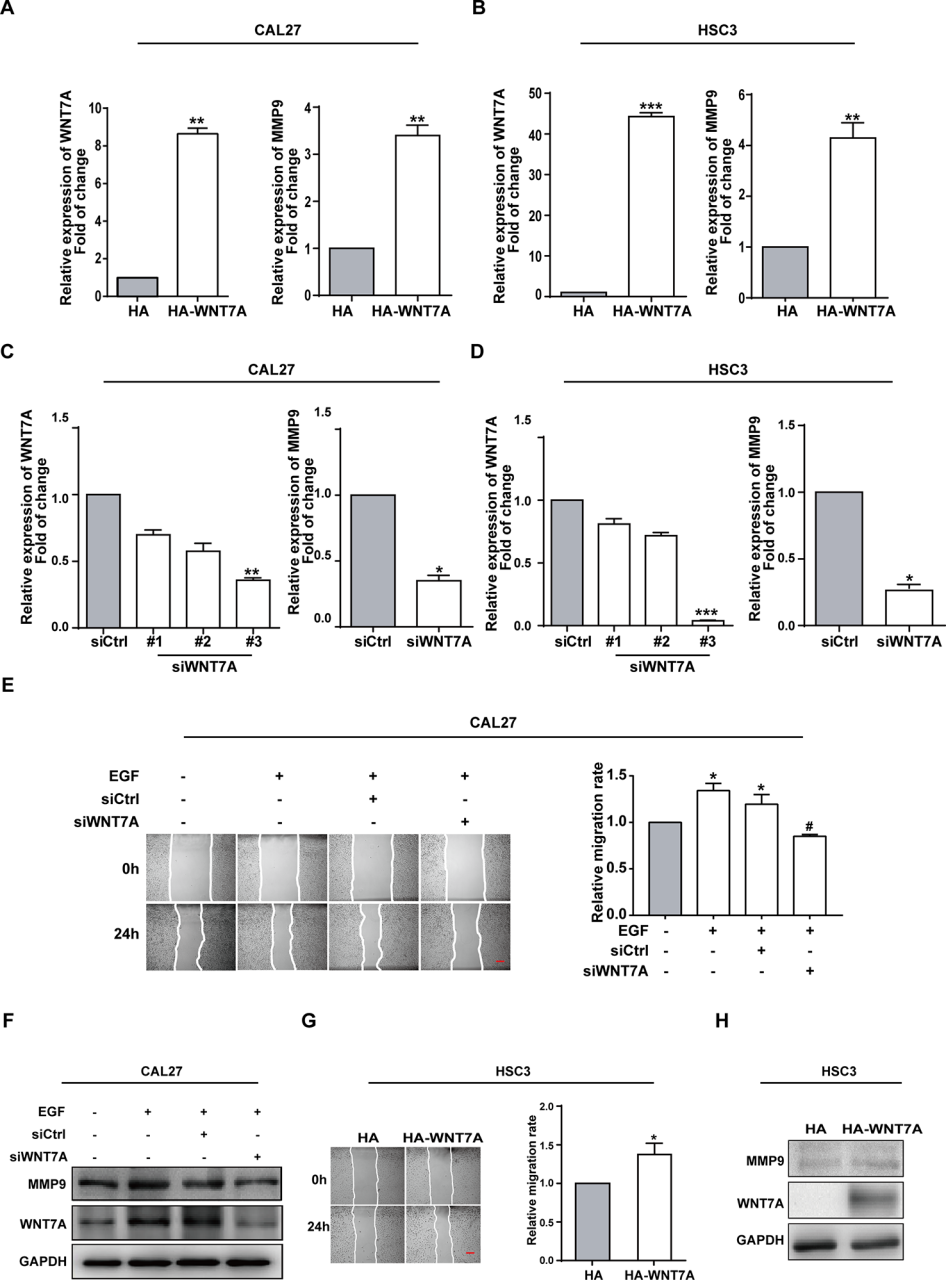
WNT7A is associated with the EGF-induced migration of OSCC cells. **(A–D)** CAL27 and HSC3 cells were transfected with empty vector, HA-tagged WNT7A, negative control siRNA, or WNT7A siRNA, respectively. qPCR was performed to detect the expression of WNT7A and MMP9. The results showed that MMP9 protein expression was changed accordingly with the expression of WNT7A. *P < 0.05, **P < 0.01, ***P < 0.001. **(E)** The wound healing assay showed that in CAL27 cells with WNT7A knockdown, incubation with EGF (20 ng/mL) did not increase the migration. Scale bar, 100 μm. *P < 0.05, in the cells incubated with EGF versus no EGF treatment; ^#^ *P* < 0.05, the siRNA-interfered WNT7A cells incubated with EGF versus the cells treated with EGF only. **(F)** The incubation with EGF did not cause the change of MMP9 protein level in CAL27 cells with WNT7A silence. **(G)** Overexpression of WNT7A induced migration of HSC3 cells. Scale bar, 100 μm. *P < 0.05. **(H)** Overexpression of WNT7A caused MMP9 expression.

The finding that EGF could induce WNT7A expression impelled us to figure out whether WNT7A was required for EGF-induced migration. The overexpression and knockdown efficiency of WNT7A in CAL27 and HSC3 cells were detected by qPCR (**Figures 2A–D**). The results showed that the MMP9 mRNA level was changed accordingly with a change in WNT7A expression. Wound healing assay was used to measure the migration of normal and WNT7A knockdown CAL27 cells incubated with or without EGF (20 ng/mL). The results showed that in CAL27 cells in which the expression of WNT7A was silenced, incubation with EGF (20 ng/mL) did not increase the migration and the expression of MMP9 (**Figures 2E, F**). Furthermore, the results of wound healing assay and Western blotting showed that the migration and MMP9 expression of HSC3 cells overexpressing WNT7A was increased (**Figures 2G, H**). The above results demonstrated that WNT7A was associated with the migration of OSCC cells induced by EGF treatment.

### EGF Regulates the Activation of the WNT7A/β-Catenin Pathway

**Figure 3:**
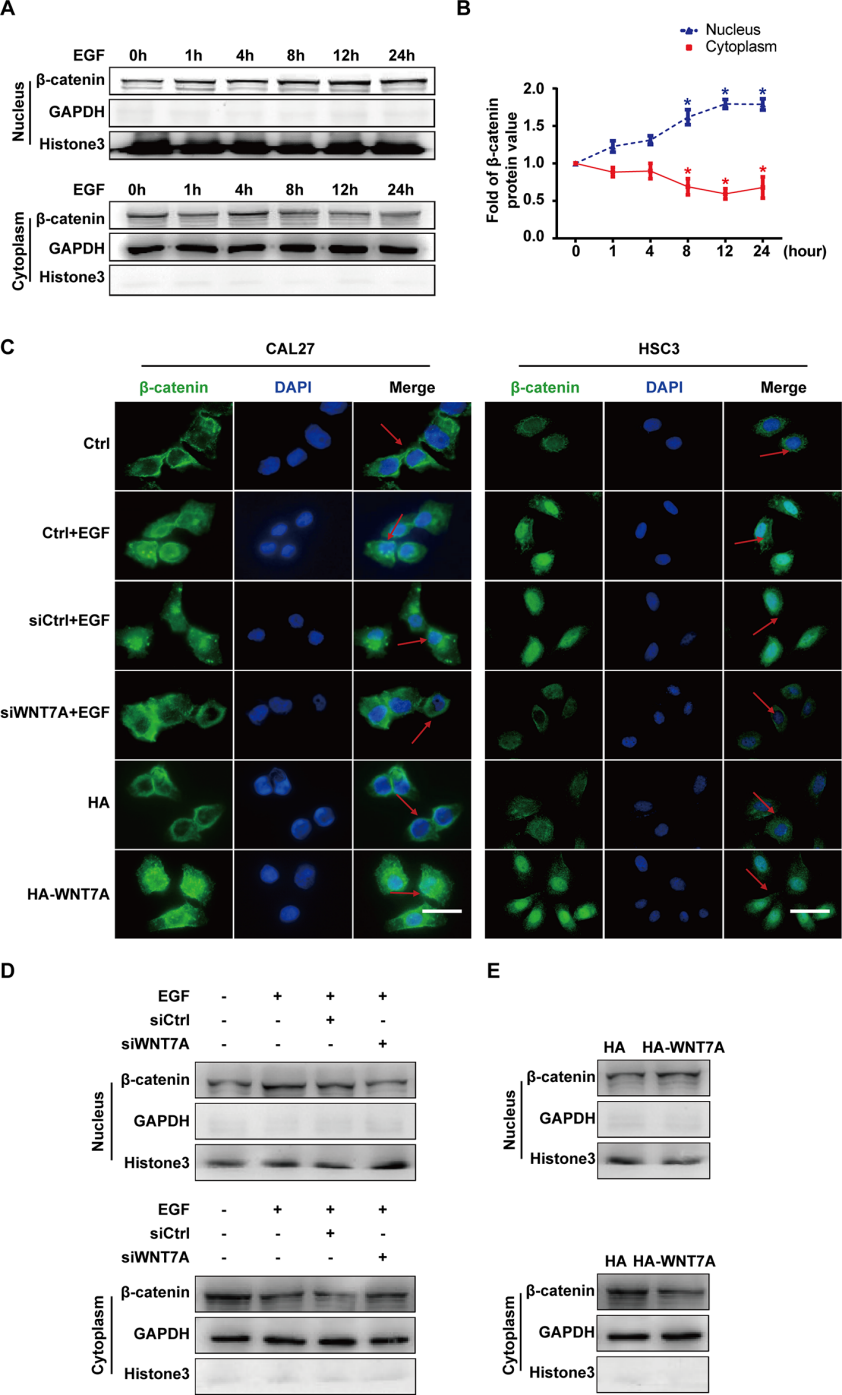
EGF regulates the activation of the WNT7A/β-catenin pathway. **(A**, **B)** CAL27 cells were incubated with EGF (20 ng/mL) for the indicated times. The results of Western blotting showed that the nuclear distribution of β-catenin increased and reached the peak level at 8-12 h with EGF treatment. ^*^ *P* < 0.05 the cytoplasm versus the nucleus. **(C)** CAL27 and HSC3 cells were divided into the indicated groups for immunofluorescent microscopy. The results showed that β-catenin accumulated in the nucleus with EGF stimulation as well as WNT7A overexpression. Knocking down *WNT7A* blocked the entrance of β-catenin into the nucleus in response to EGF stimulation. Scale bar, 10 μm. **(D)** In CAL27 cells incubated with 20 ng/mL EGF, the protein level of β-catenin in the nucleus was decreased by *WNT7A* silence. **(E)** β-catenin accumulated in the nuclei of CAL27 cells overexpressing WNT7A.

Considering that β-catenin was the classical downstream signaling molecule of WNT7A, we investigated whether the WNT7A/β-catenin pathway was activated by EGF stimulation. The EGF-induced expression and distribution of β-catenin in the nucleus and cytoplasm were examined, respectively. The Western blotting results showed that the nuclear distribution of β-catenin increased and reached the peak level with EGF treatment for 8-12 h in CAL27 cells (**Figures 3A, B**). The results of confocal microscopy showed that the knockdown of WNT7A in CAL27 and HSC3 cells blocked the accumulation of β-catenin in the nucleus in response to EGF stimulation. Conversely, after transfection with WNT7A plasmids, β-catenin accumulated in the nucleus (**Figure 3C**). Furthermore, Western blotting results showed that the distribution of β-catenin in the nucleus was decreased by the silencing of WNT7A expression when CAL27 cells were incubated with 20 ng/mL EGF (**Figure 3D**). Also, the nuclear distribution of β-catenin was increased in CAL27 cells overexpressing WNT7A (**Figure 3E**). Therefore, the above results suggested that WNT7A was involved in the process of EGF-activated Wnt/β-catenin signaling.

### EGF Induces Cell Migration *via* MEK/ERK and PI3K/AKT Signalings, but Only p-AKT is Involved in the Wnt/β-Catenin Pathway

**Figure 4:**
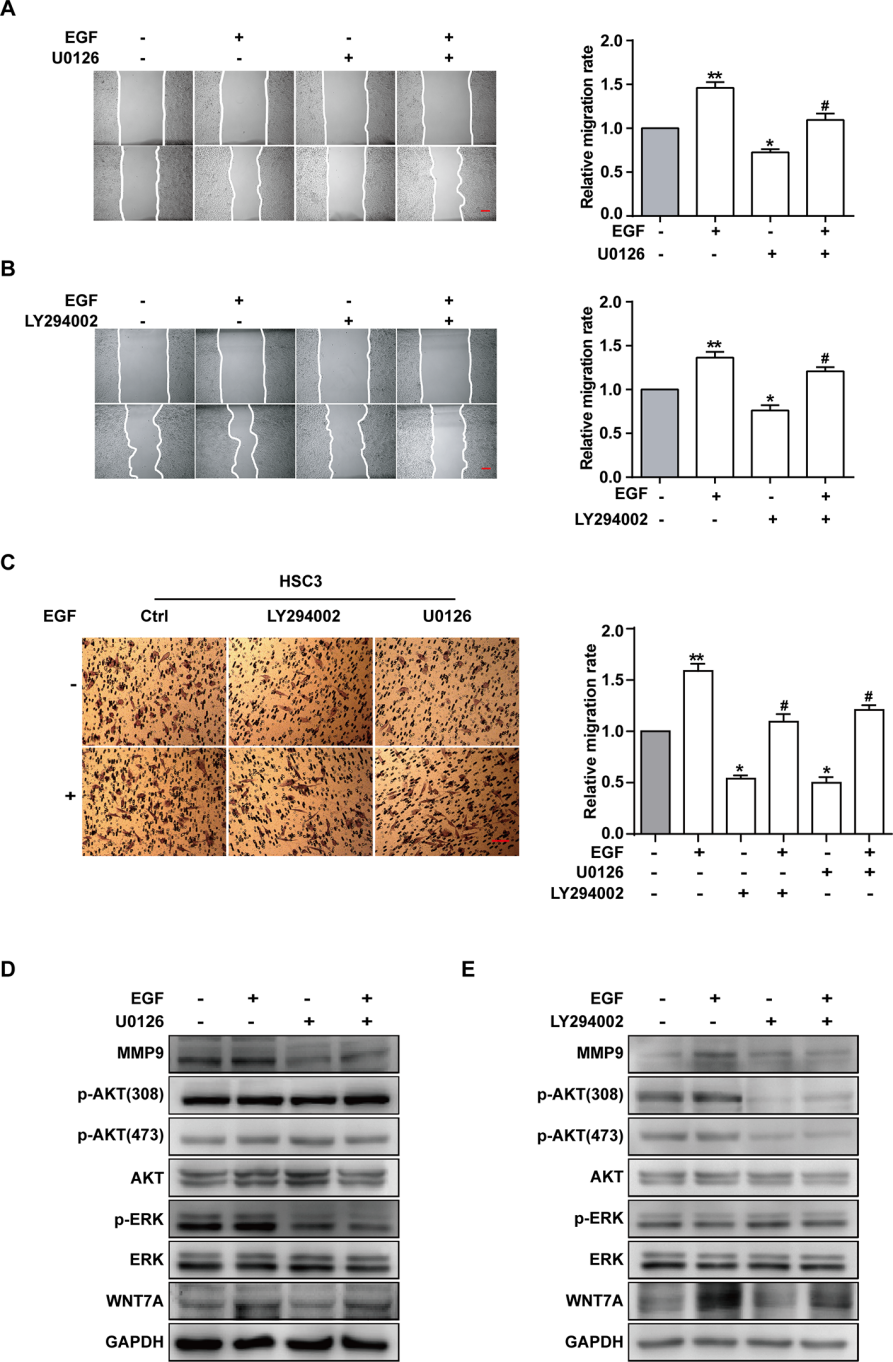
EGF induces cell migration *via* MEK/ERK and PI3K/AKT signalings, but only p-AKT is involved in the Wnt/β-catenin pathway. **(A–E)** CAL27 cells were incubated for 2 h in the absence or presence of 10 μM U0126 or 10 μM LY294002 prior to EGF treatment (20 ng/mL) for 24 h. The wound healing assay showed that pre-treatment with MEK inhibitor U0126 **(A)** and PI3K/AKT inhibitor LY294002 **(B)** could reverse the EGF-induced migration. *P < 0.05, **P < 0.01 in the cells treated with EGF combined with U0126 or LY294002 versus the cells in the control group. ^#^ *P* < 0.05 in the cells incubated with EGF combined with U0126 or LY294002 versus the cells treated with EGF only. Scale bar, 100 μm. **(C)** Transwell assay showed that EGF (20 ng/mL) treatment induced the migration of HSC3 cells and that pre-treatment with U0126 and LY294002 could reverse the process. *P < 0.05, **P < 0.01, in the cells treated with EGF combined with U0126 or LY294002 versus the cells in the control group. ^#^ *P* < 0.05, in the cells incubated with EGF combined with U0126 or LY294002 versus the cells treated with EGF only. **(D)** The Western blotting results indicated that U0126 could down-regulate MMP9 expression but had no significant effect on the p-AKT or WNT7A level. **(E)** The Western blotting results showed that LY290042 could down-regulate WNT7A and MMP9 expression but had no significant effect on the p-ERK level.

ERK and PI3K/AKT are critical components of EGF-activated signaling, which has been associated with human cancer EMT (Wudtiwai et al., 2018; Jiang et al., 2019; Park et al., 2019; Yang et al., 2019; Yoo et al., 2019). To verify whether ERK and PI3K/AKT could affect EGF-mediated migration of CAL27 cells, MEK inhibitor U0126 and PI3K/AKT inhibitor LY294002 were applied in observing the cell migration in the absence or presence of EGF (20 ng/mL) for 24 h. The results showed that pre-treatment with U0126 and LY294002 could reverse the EGF-induced migration (**Figures 4A, B**). Transwell assay was carried out in HSC3 cells to verify the effect of U0126 and LY294002 on the migration (**Figure 4C**). We found that the migration of HSC3 cells was noticeably increased by EGF (20 ng/mL) stimulation. Besides, pre-treatment with both U0126 and LY294002 could reverse the EGF-induced migration.

Furthermore, the result of Western blotting indicated that treatment with U0126 and LY290042 could down-regulate MMP9 expression (**Figures 4D, E**). Interestingly, in cells pre-treated with U0126 and incubated with or without EGF, the level of p-AKT and WNT7A remained consistent (**Figures 4D, E**). In addition, we noticed that LY290042 could down-regulate WNT7A expression but had no significant effect on the p-ERK level (**Figures 4D, E**). Furthermore, qPCR was applied to detect the WNT7A mRNA expression in the absence or presence of U0126 and LY294002 with or without EGF stimulation in both CAL27 cells and HSC3 cells (**Figures S2A, B**). Consistent with the Western blotting results, treatment with LY294002 rather than U0126 affected the expression of WNT7A mRNA.

The above results suggested that both MEK/ERK and PI3K/AKT contributed to the EGF-induced migration, while p-AKT was involved in the EGF-activated WNT7A/β-catenin signaling.

### EGF Steers a Two-Pronged (MEK/ERK and PI3K/AKT) Pathway Toward Migration

**Figure 5:**
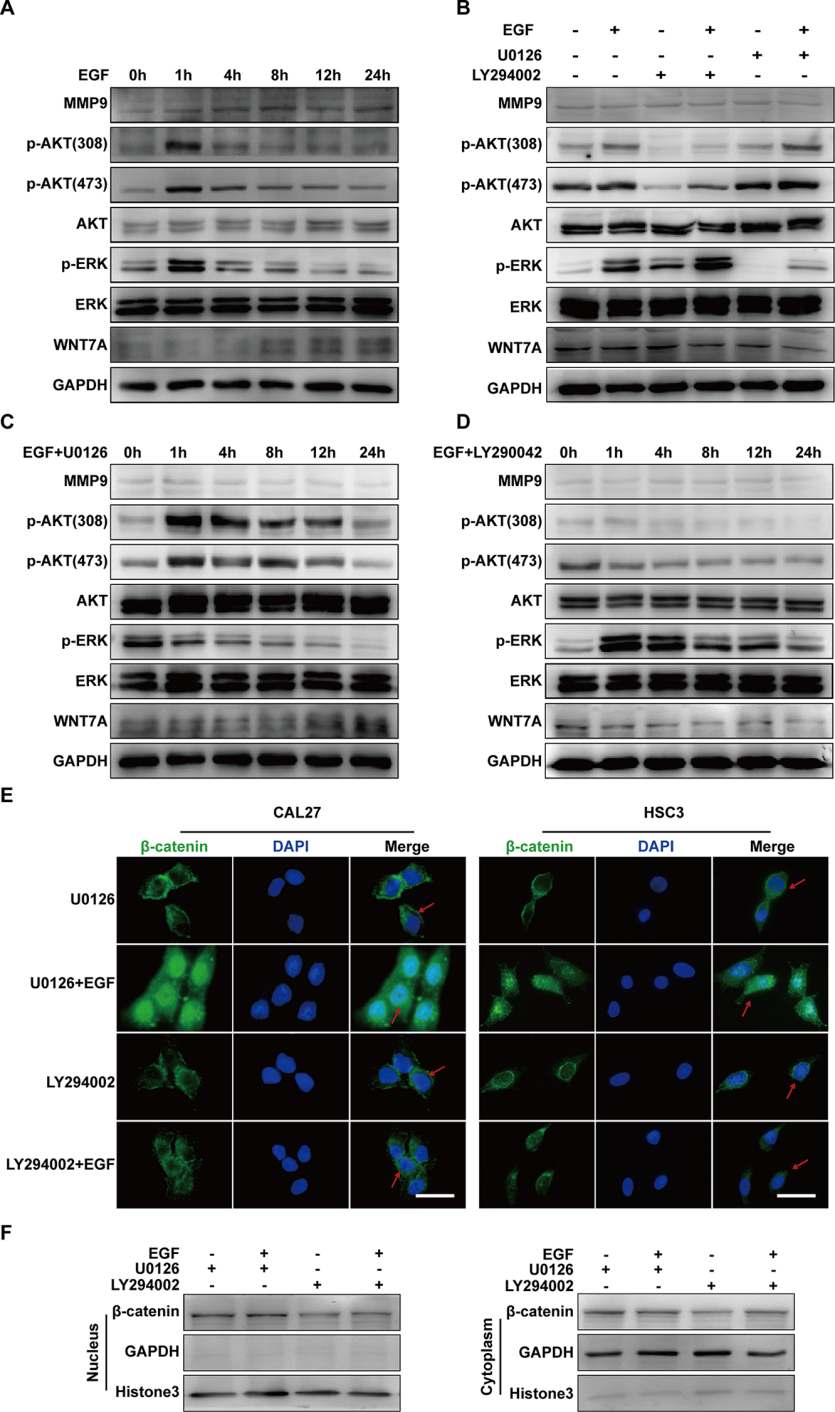
EGF steers a two-pronged (MEK/ERK and PI3K/AKT) pathway toward migration. **(A)** CAL27 cells were incubated in EGF (20 ng/mL) for the indicated times, and the results of Western blotting showed that p-AKT (Thr308), p-AKT(Ser473), and p-ERK reached their peak levels at 1 h with EGF stimulation. **(B)** Cells were treated with a different combination of EGF with U0126 or LY294002 for 1 h. The results of Western blotting showed that U0126 did not affect the phosphorylation of AKT and that LY290042 did not affect the phosphorylation of ERK either. **(C, D)** The results of Western blotting showed that in cells with pretreatment of U0126 **(C)** or LY294002 **(D)** and treatment with EGF for the indicated times, MEK/ERK and PI3K/AKT did not affect each other. Also, PI3K/AKT inhibitor but not MEK/ERK inhibitor had an effect on WNT7A expression. The results of immunofluorescence microscopy **(E)** and Western blotting **(F)** showed that pretreatment with U0126 did not prevent EGF-induced β-catenin accumulation in the nucleus. In contrast, pretreatment with LY294002 blocked the accumulation induced by EGF. Scale bar, 10 μm.

The investigation was carried out into whether there was crosstalk between MEK/ERK and PI3K/AKT signalings in EGF-induced migration. Firstly, to probe the activation of ERK and AKT during EGF-induced migration, the levels of p-AKT (Thr308), p-AKT(Ser473), p-ERK, MMP9, and WNT7A in cells treated with EGF for the indicated periods were detected. The results showed that p-AKT (Thr308), p-AKT (Ser473), and p-ERK reached their peak levels at 1 h with EGF stimulation (**Figure 5A**). Next, to verify the relation between p-ERK and p-AKT, their levels in CAL27 cells incubated with U0126 or LY294002 for 1 h in the presence or absence of EGF were detected. As shown in **Figure 5B**, U0126 did not affect the phosphorylation of AKT, and LY290042 did not affect the phosphorylation of ERK. Furthermore, the results confirmed that LY290042 but not U0126 had an effect on WNT7A expression (**Figures 5C, D**).

Since the above results suggested that both p-ERK and p-AKT might regulate the migration of CAL27 cells and that WNT7A could be down-regulated by LY290042, we hypothesized that p-AKT might be involved in the regulation of WNT7A. To further explore whether and how p-ERK and p-AKT mediate the EGF-activated signaling, immunofluorescence microscopy and Western blotting were carried out to detect the distribution of β-catenin. The results showed that pre-treatment with U0126 and EGF could induce β-catenin accumulation in the nucleus, while pre-treatment with LY294002 and EGF did not cause its accumulation (**Figures 5E, F**). Therefore, these results demonstrated that p-AKT rather than p-ERK was involved in the EGF-activated Wnt/β-catenin signaling.

**Figure 6:**
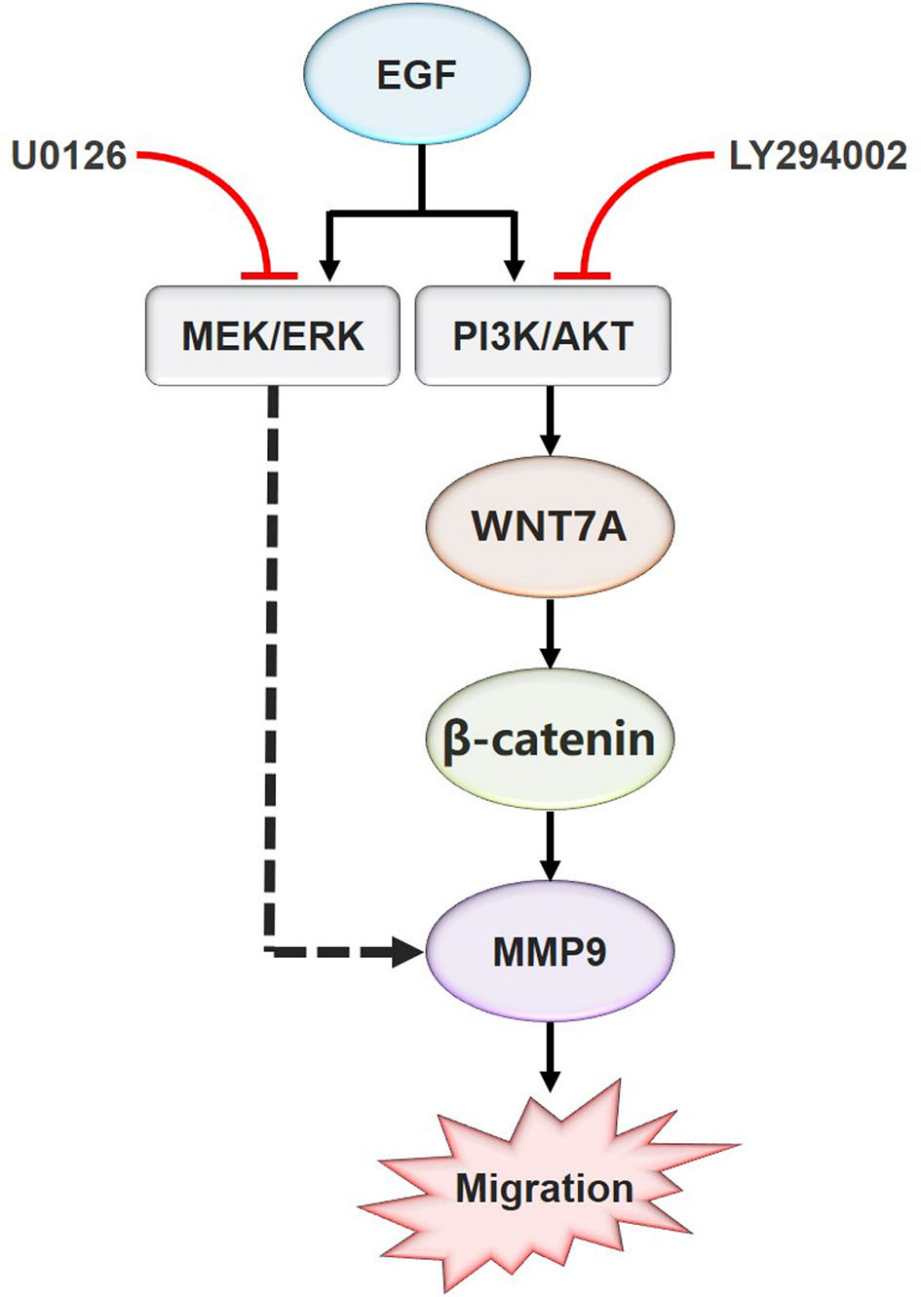
Schematic model for the EGF-mediated migration regulation. In summary, the present study showed that WNT7A mRNA and protein levels were increased by EGF stimulation. In addition, the study also proved that p-AKT, but not p-ERK, mediated the expression of WNT7A protein induced by EGF. Furthermore, inhibition of AKT activation prevented the upregulation of WNT7A expression, the translocation of β-catenin from the cytoplasm to the nucleus, and the increase of MMP9 expression induced by EGF. Moreover, EGF activates ERK at the same time, which directly targets MMP9 to promote the migration of OSCC cells.

In summary, these results indicated that EGF steers a two-pronged (MEK/ERK and PI3K/AKT) pathway toward migration. On the one hand, EGF treatment could increase the phosphorylation of AKT, causing the nuclear accumulation of β-catenin, the increase of WNT7A and MMP9 expression, and the stimulation of migration in CAL27 cells. On the other hand, EGF activated ERK at the same time, which could target MMP9 directly to promote the migration (**Figure 6**).

### Expression of WNT7A Protein in OSCC Correlates With the Differentiation of the OSCC Cells

**Figure 7:**
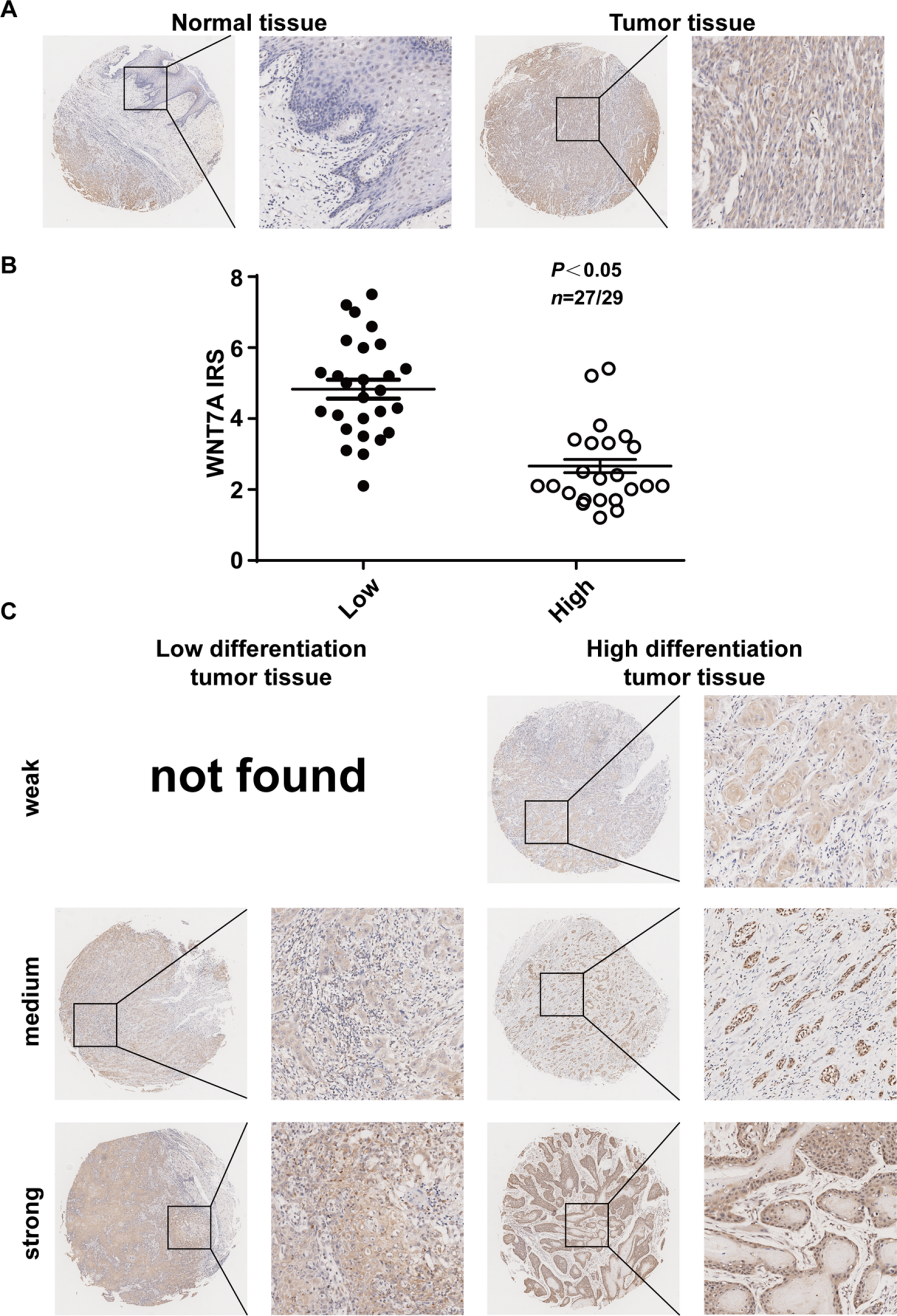
Expression of WNT7A protein in OSCC tissues correlates with the differentiation of the cells. **(A)** Immunostaining of cancer tissue and its adjacent tissue indicated that WNT7A was highly expressed in tumor tissues compared with matched normal tissues. Brown, WNT7A; Blue, hematoxylin. **(B)** IRS scores of WNT7A according to tumor histological grade. P values and tissue samples are shown above the scatter diagram. **(C)** Immunostaining of high and low differentiated OSCC tissues showed that the WNT7A expression was markedly increased in poorly differentiated tumor tissues compared with well-differentiated tumor tissues.

To explore whether our *in vitro* experimental results were consistent with the pathogenesis of OSCC, we examined the expression of WNT7A in OSCC tissue and its adjacent tissue (five paired cases) as well as high and low differentiated OSCC specimens (56 cases). The representative immunohistochemistry results are shown in **Figure 7**. The results indicated that the expression of WNT7A in tumor tissues was higher than that in matched adjacent tissues (**Figure 7A**). Among 56 OSCC cases, the WNT7A expression in poorly differentiated tumor tissues was markedly increased compared with the expression in well-differentiated tumor tissues (**Figures 7B, C**). Overall, the clinical data supported our *in vitro* results in indicating that WNT7A might play a key role in OSCC progression.

## Discussion

OSCC is one of the most common malignant tumors of the head and neck area (Chundayil Madathil et al., 2019; Feng et al., 2019). Although the morbidity and mortality of OSCC have gradually decreased recently, the 5-year survival rate remains lower than 50% (Torre et al., 2016; Bray et al., 2018). It is widely accepted that high rates of lymph node metastasis and distant metastasis are the major causes of OSCC-related death (Huang et al., 2008; Ganly et al., 2012; D’Cruz et al., 2015). Therefore, elucidating potential mechanisms that regulate OSCC metastasis is critical for the treatment of OSCC. However, the molecular mechanisms underlying the metastasis in OSCC were not fully understood. In this paper, it was revealed that WNT7A was involved in the EGF-induced migration of OSCC cells, and the related mechanism was also elucidated. This will be beneficial for the understanding of the metastasis mechanisms of OSCC.

The existing research data about the association of WNT7A with cancers are inconsistent. For example, research results obtained from NSCLC indicated that WNT7A was a tumor suppressor and that its expression was low in NSCLC tissues (Kondratov et al., 2012; Avasarala et al., 2013; Bikkavilli et al., 2015). However, research results from ovary cancer demonstrated that WNT7A could promote the process of the tumor (Huang et al., 2014; Park et al., 2015). This contradiction suggested that the role of WNT7A in tumorigenesis was type-dependent and that investigation of its role in more cancers was needed. Here, in this paper, our results confirmed that WNT7A expression was markedly increased in poorly differentiated tumor tissues compared with matched well-differentiated tumor tissues and that EGF could cause an increase of WNT7A expression in OSCC cells. The results provided a further experimental basis for us to recognize the multiple roles of WNT7A in mutagenesis.

In the present study, we examined the mRNA expression of all members of the *Wnt* gene family in CAL27 and HSC3 cells. We found that WNT7A was abundantly expressed in the CAL27 cells and that WNT5A was abundantly expressed in the HSC3 cells. This difference may relate to the characteristics of different cell lines. Previous studies already proved that WNT5A acts as a regulator of OSCC *via* non-canonical Wnt signaling (Prgomet et al., 2015; Sakamoto et al., 2017). Therefore, our research focused on the effect of WNT7A on OSCC progression.

In this paper, the role of WNT7A in the EGF-induced migration of OSCC cells was explored step by step. First, since recent studies revealed that increased MMP9 expression was associated with the increase of cell migration activity in response to chemical stressors (Huang et al., 2014; Park et al., 2015; Wang et al., 2018; Zhang et al., 2018; Xue et al., 2019), the relations among EGF, WNT7A, and MMP9 were investigated. The results showed that WNT7A mediated the EGF-induced migration and MMP9 expression: the knockdown of WNT7A reversed the EGF-induced increase in migration activity, and the overexpression of WNT7A enhanced the migration and the expression of MMP9. Considering that WNT7A could regulate cancer progression through canonical or non-canonical Wnt signaling (Bikkavilli et al., 2015; Huang et al., 2018), our subsequent studies were focused on the downstream signaling of WNT7A in response to EGF stimulation. It was found that EGF-induced activation of WNT7A was accompanied by β-catenin accumulation in the nucleus. Consistently, the process of EGF-induced β-catenin accumulation was blocked by silencing WNT7A. This suggested that the reversal of EGF-induced β-catenin accumulation in the nucleus was associated with WNT7A expression. Meanwhile, overexpression of WNT7A could induce β-catenin accumulation in the nucleus. Thus, our data indicated that EGF-induced migration of OSCC cells was associated with the activation of the WNT7A/β-catenin pathway. Accordingly, we speculated that WNT7A played a critical role *via* canonical Wnt signaling in the regulation of OSCC cell migration.

Given our observation that EGF especially stimulated WNT7A expression at mRNA and protein levels, it would be meaningful to elucidate how EGF regulates the expression of WNT7A. The previous study reported that MEK/ERK and PI3K/AKT could regulate EGFR signaling (Li et al., 2019). We hypothesized that the two pathways might also be involved in the EGF-specific regulation of WNT7Aexpression in OSCC cells and therefore investigated their role in the regulation. We proved that EGF induced a time-dependent increase in ERK and AKT phosphorylation, which happened before the increase in WNT7A and MMP9 expression. Additionally, when MEK/ERK and PI3K/AKT signalings were blocked, EGF-induced migration was dramatically diminished, suggesting that both of them played a role in the process.

Interestingly, we also found that EGF-induced increase of the expression of WNT7A and MMP9 was dependent on AKT activation and that inhibition of AKT phosphorylation but not ERK phosphorylation could reverse the EGF-induced WNT7A expression and β-catenin accumulation in the nucleus. Therefore, we speculated that the phosphorylation of AKT might affect WNT7A expression *via* the canonical Wnt/β-catenin pathway. Intriguingly, our results also revealed that ERK might regulate EGF-induced migration by directly targeting MMP9, independent of WNT7A. Hence, our results indicate that the PI3K/AKT but not the MEK/ERK pathway is involved in the EGF-induced WNT7A/β-catenin signaling.

Besides, the FZD family is the most crucial receptor family in the Wnt/β-catenin pathway, and abnormal expression of FZDs is closely related to carcinogenesis. In our study, we found that FZD3 and FZD6 were highly expressed in CAL27 and HSC3 cells. A recent study revealed that the FZD3-mediated Wnt/β-Catenin signaling pathway was activated in breast cancer cells (Mo et al., 2019). Other studies indicated that FZD6 contributes to the metastasis of colorectal cancer and the malignancy of acute myeloid leukemia cells through activating Wnt/β-catenin signaling (Xu et al., 2019; Yuan et al., 2019). Combining our results with the previous research data, we hypothesize that how the cells respond to WNT7A may depend on the FZD receptor that is expressing.

In summary, the results in this paper confirm that EGF induces the expression of WNT7A *via* activating AKT and that WNT7A/β-catenin signaling increases the expression of MMP9. Both of them enhance the migration of OSCC cells. These findings suggest that WNT7A is a promoter of the progression of OSCC, having potential pathophysiological importance for the study of OSCC, and providing new insights for the therapeutic targets of this cancer.

## Data Availability Statement

The raw data supporting the conclusions of this article will be made available by the authors, without undue reservation, to any qualified researcher.

## Author Contributions

FZ, LG, YZ, and HX designed the study. HX, YM, ST, and SZ completed the experiments. JL, HC, YX, MC performed the statistical analysis. XZ, YZ, and JD drafted the manuscript and supervised the experimental work. All authors read and approved the final manuscript.

## Funding

This study was supported by the National Key Research and Development Project of China (Grant number 2016YFA0201704/2016YFA0201700), the Priority Academic Program Development of Jiangsu Higher Education Institutions (Grant number 2018-87), and the National Natural Science Foundation of China (Grant number 81602561, 81773107).

## Conflict of Interest

The authors declare that the research was conducted in the absence of any commercial or financial relationships that could be construed as a potential conflict of interest.

## Supplementary Material

Figure S1 FZDs, LRP, EGFR, MMP9, and WNT7A expression in CAL27 and HSC3 cells. **(A–C)**. The results of qPCR showed that FZDs, LRP, EGFR, MMP9, and WNT7A were highly expressed in the OSCC cells. Western blotting results showed that in CAL27 cells, the EGFR, MMP9, and WNT7A levels were higher than in HSC3 cells **(D)**.

Figure S2 WNT7A mRNA expression after EGF treatment in the absence or presence of U0126 and LY294002. The results showed that treatment with LY294002 rather than U0126 had an effect on WNT7A mRNA expression in both CAL27 and HSC3 cells **(A, B)**. **P* < 0.05, ***P* < 0.01, in the cells treated with EGF combined with U0126 or LY294002 versus the cells in the control group. #*P* < 0.05, in the cells treated with EGF combined with U0126 or LY294002 versus the cells treated with EGF only.
