# Supplementary figures and images for: WNT7A Promotes EGF-Induced Migration of Oral Squamous Cell Carcinoma Cells by Activating β-Catenin/MMP9-Mediated Signaling
Source: Front Pharmacol. 2020 Feb 26;11:98. doi: 10.3389/fphar.2020.00098 (PMC7054863; doi:10.3389/fphar.2020.00098)

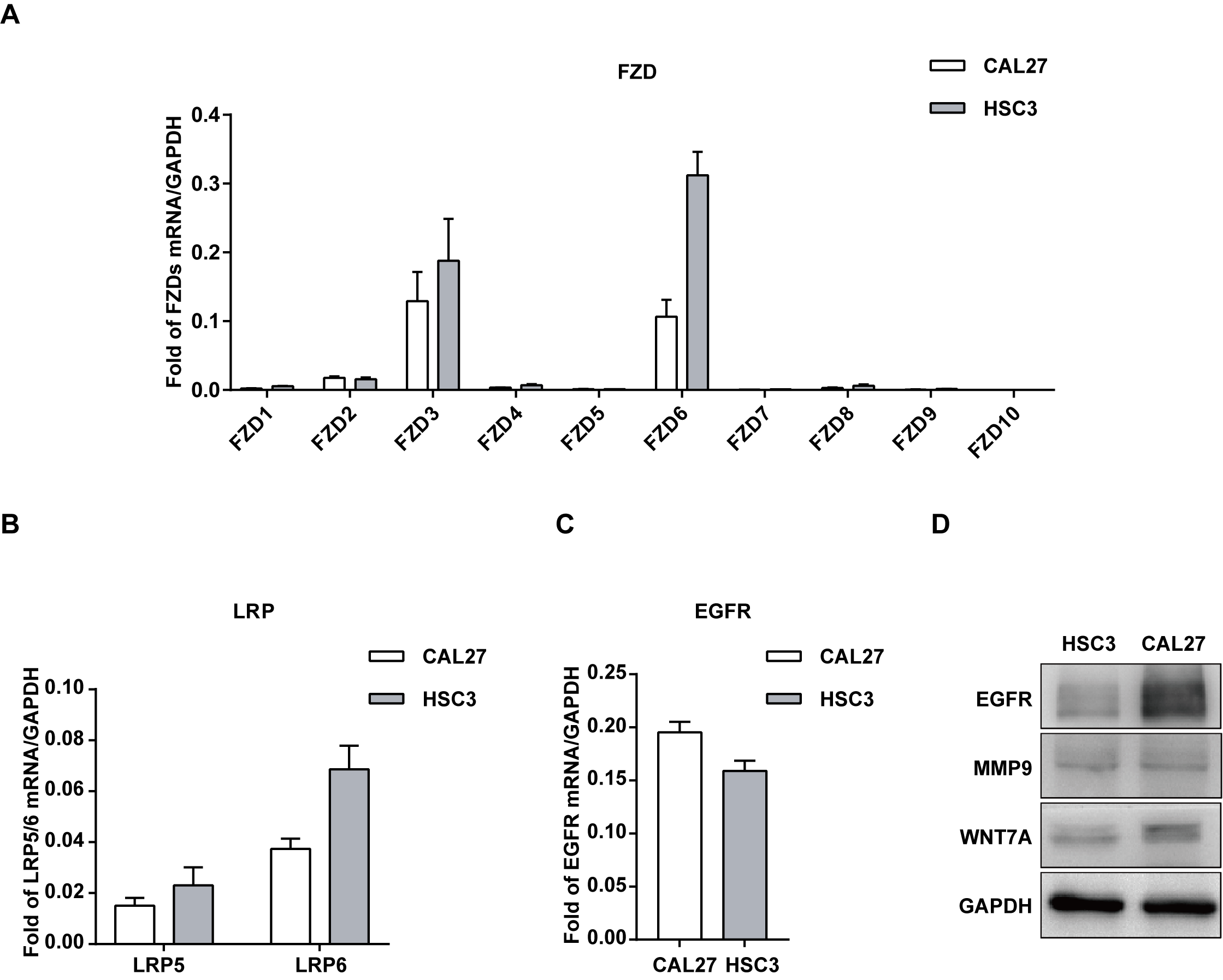

Supplement: Figure S1 — FZDs, LRP, EGFR, MMP9, and WNT7A expression in CAL27 and HSC3 cells. (A–C). The results of qPCR showed that FZDs, LRP, EGFR, MMP9, and WNT7A were highly expressed in the OSCC cells. Western blotting results showed that in CAL27 cells, the EGFR, MMP9, and WNT7A levels were higher than in HSC3 cells (D). [file Image_1.tif]

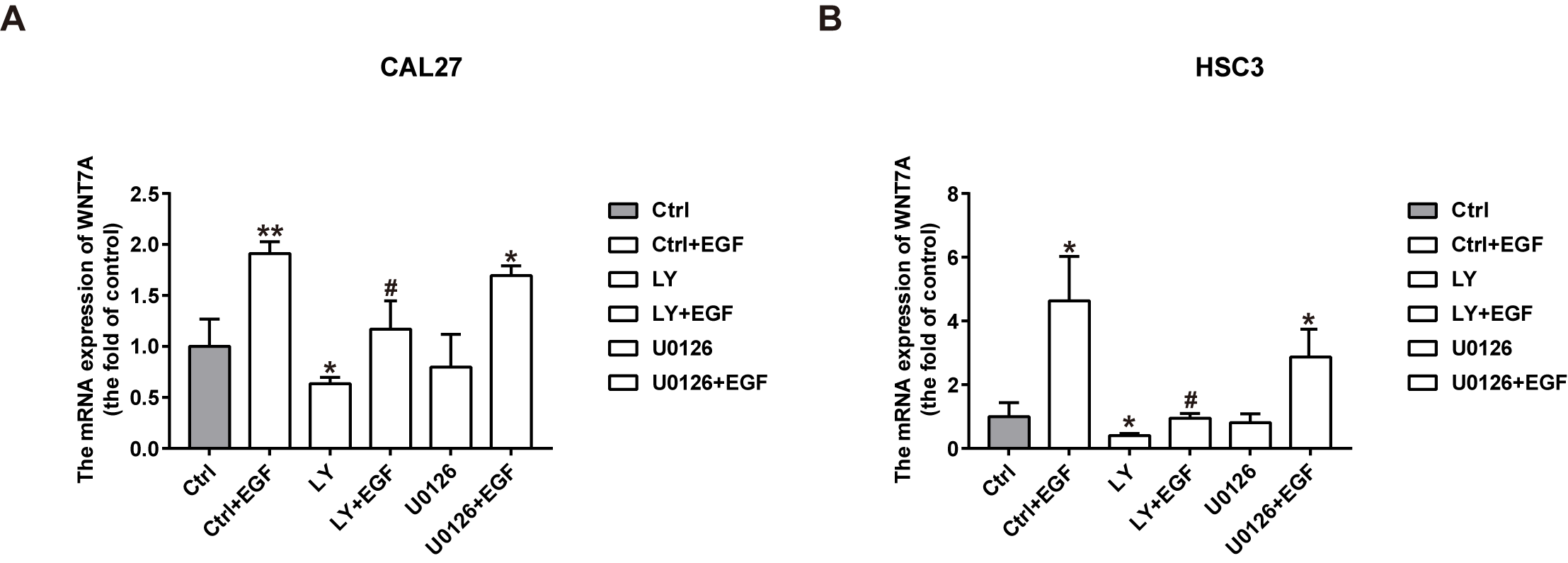

Supplement: Figure S2 — WNT7A mRNA expression after EGF treatment in the absence or presence of U0126 and LY294002. The results showed that treatment with LY294002 rather than U0126 had an effect on WNT7A mRNA expression in both CAL27 and HSC3 cells (A, B). *P < 0.05, **P < 0.01, in the cells treated with EGF combined with U0126 or LY294002 versus the cells in the control group. #P < 0.05, in the cells treated with EGF combined with U0126 or LY294002 versus the cells treated with EGF only. [file Image_2.tif]
